# Secrecy Enhancing Scheme for Spatial Modulation Using Antenna Selection and Artificial Noise

**DOI:** 10.3390/e21070626

**Published:** 2019-06-26

**Authors:** Pingping Shang, Weicheng Yu, Kai Zhang, Xue-Qin Jiang, Sooyoung Kim

**Affiliations:** 1 Division of Electronic Engineering, IT Convergence Research Center, Chonbuk National University, Jeonju 54896, Korea; 2 Shanghai Aerospace Electronic Technology Institute, Shanghai 201109, China; 3 Department of Communication Engineering, Donghua University, Shanghai 201620, China

**Keywords:** spatial modulation (SM), antenna selection, artificial noise (AN), channel state information (CSI), physical layer security (PLS), secrecy rate

## Abstract

In this paper, we present a new secrecy-enhancing scheme for the spatial modulation (SM) system, by considering imperfect channel state information (CSI). In the proposed scheme, two antennas are activated at the same time. One of the activated antennas transmits information symbols along with artificial noise (AN) optimized under the imperfect CSI condition. On the other hand, the other activated antenna transmits another AN sequence. Because the AN are generated by exploiting the imperfect CSI of the legitimate channel, they can only be canceled at the legitimate receiver, while the passive eavesdropper will suffer from interference. We derive the secrecy rate of the proposed scheme in order to estimate the performance. The numerical results demonstrated in this paper verify that the proposed scheme can achieve a better secrecy rate compared to the conventional scheme at the same effective data rate.

## 1. Introduction

Spatial modulation (SM) is a spatial multiplexing multiple-input multiple-output (MIMO) scheme, and it is known that SM can overcome the drawbacks of the conventional MIMO techniques [1]. For example, SM can entirely avoid the inter-channel interference (ICI) and inter-antenna synchronization (IAS) problems by activating only a part, usually one of the transmit-antennas for data transmission at any signaling time instance. In addition, it usually needs one radio frequency (RF) chain for data transmission [2]. Due to the above-mentioned advantages, SM is expected to be one of the key technologies for future wireless networks [3].

SM is considered as a three-dimensional (3D) modulation technique, because it conveys information bits by utilizing both the antenna index and complex symbols to form a 3D constellation [4]. With this concept, efficient 3D constellation schemes were proposed in order to achieve a better spatial multiplexing gain and high data throughput [4,5]. It was emphasized that they could be easily extended to a massive MIMO system by reducing the cost of the massive numbers of RF chains at the base stations, and the security scheme could be directly adopted. On the other hand, efficient antenna selection techniques were proposed to enhance the system performance. For example, a Euclidean distance optimized antenna selection (EDAS) method was proposed to offer a significant signal-to-noise ratio (SNR) gain [6], and later, the achievable transmit diversity order of the EDAS was analyzed [7].

Due to the inherent broadcasting nature of wireless communication systems, security and privacy protection comprise an increasingly important issue in the design and implementation of wireless networks. Recently, physical layer security (PLS) schemes combined with MIMO techniques have gained much attention, especially when SM is used [8]. Because SM changes the active antenna dynamically, it can be easily applied to a PLS scheme. For example, an antenna selection scheme for SM was proposed to achieve PLS transmission [9].

In combination with the antenna selection, the idea of injecting jamming signals, i.e., artificial noises (AN), into the transmission of SM was proposed to enhance the security [10]. It was presented that activating an extra transmit antenna to transmit AN can increase the security of SM. A similar fact was discussed in [11], where the authors proposed a secure transmission scheme for differential quadrature spatial modulation (DQSM) with AN, and it was shown that the AN technique was a meaningful idea to improve the secrecy performance. Because the AN can be also interference to the legitimate receiver, the antenna carrying AN needs to be directed to the null space, and thus, additional beamforming technique was required. One of our previous studies presented a PLS scheme with AN, which did not require a beamforming technique [12]. On the other hand, a symbol rotation-based secrecy-enhancing scheme combined with SM was proposed [13]. In this scheme, complex symbol values for transmit antenna indices and information were dynamically rotated, where the rotation values were optimized for legitimate channel state information (CSI).

However, all of the aforementioned schemes assumed perfect CSI knowledge at least for the legitimate channel. By considering that the influence of imperfect CSI due to the channel estimation error cannot be ignored, this paper proposes a new secrecy-enhancing scheme for SM under imperfect CSI. We adopt the idea of combining the antenna selection with AN for PLS and utilize the additional activation of the antenna only for transmitting AN. Especially, we propose a method to employ two AN sequences, which have the co-cancellation property, and thus, they can perfectly cancel each other only at the legitimate receiver. We assume the imperfect CSI condition by considering the practical system operation condition, and thus, the generation of AN and antenna selection are all optimized by considering imperfect CSI. In this way, the legitimate receiver can perfectly cancel the AN while the eavesdropper will suffer from the interference induced by the AN. To estimate the secrecy performance, we derive the secrecy rate of the proposed scheme.

The rest of this paper is organized as follows. In Section 2, we describe the basic concept of the conventional PLS scheme with SM by using a simple system model. Section 3 presents the proposed scheme, starting with the representation of the operational principle in brief. Afterwards, it proposes a method to generate AN, which can be perfectly canceled only at the legitimate receiver, and then presents the optimum antenna selection method by considering imperfect CSI. In Section 4, we derive the mathematical expressions of the secrecy rate of the proposed scheme. The numerical results and discussions are presented in Section 5. Finally, Section 6 draws the conclusions.

Notation: Bold lower case letters represent vectors, while bold upper case letters denote matrices. ${\left(\cdot \right)}^{H}$, |·|, and ∥·∥ denote the Hermitian transpose, modulus operator, and Frobenius norm operations, respectively. ·· is the binomial coefficient. $C^{m\times n}$ stands for the complex space of m×n dimensions.

## 2. PLS Scheme with SM

**Figure 1:**
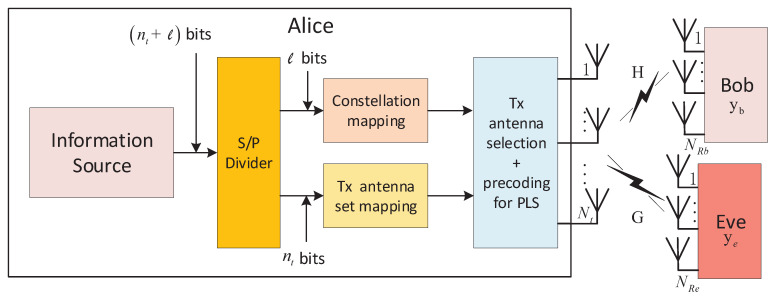
Physical layer security scenario for the spatial modulation systems.

Figure 1 shows a system model using SM for PLS. In the generic model of the PLS scheme, there is a cooperative wireless network consisting of three nodes, as shown in Figure 1. One source node, which is the legitimate transmitter node, is referred to as Alice. The corresponding destination node is referred to as Bob, which is the legitimate receiver node. On the other hand, the third node, named Eve, is the passive eavesdropper node. In this model, Alice sends the secret data sequence and communicates confidentially with Bob. Eve attempts to intercept the ongoing communication between the legitimate link, i.e., from the transmitter Alice to the legitimate receiver Bob. In other words, Eve tries to decode and obtain the secret data content from her own observations of the received signals [9].

As shown in Figure 1, it is assumed that the transmitter Alice is equipped with $N_{t}$ antennas, and *M*-ary amplitude and phase modulation (APM) symbols are transmitted over one of the $N_{t}$ antennas at each signaling time instance. The channels from Alice to Bob and Eve are represented by the $N_{Rb}\times N_{t}$ matrix **H** and $N_{Re}\times N_{t}$ matrix **G**, respectively, where $N_{Rb}$ and $N_{Re}$ are the numbers of receive antennas at Bob and Eve, respectively. All the channel gains for each transmitted symbol, i.e., all the elements of **H** and **G**, are assumed to be independent complex Rayleigh random variables and not to have frequency selectivity. The number of transmit antennas, $N_{t}$, in the conventional SM system is usually assumed to be a power of two [1]. At the transmitter, the bit stream emitted by a binary source is divided into blocks containing $n_{t}$ and *ℓ* bits each, where $n_{t}=\log_{2}N_{t}$ is the number of bits to identify a transmit-antenna among $N_{t}$ antennas, and $\ell =\log_{2}M$ is the number of bits in an *M*-ary symbol. Therefore, the effective data rate $r_{e}=n_{t}+\ell$.

When a symbol is chosen to be transmitted at the *j*-th antenna among $N_{t}$ antennas, the transmission from Alice can be represented by the $N_{t}\times 1$ column vector, **x**, as follows [14]:

(1) $$x={\left[0\ldots 0\underset{j-th}{\underbrace{x_{m}}}0\ldots 0\right]}^{T}=e_{j}\cdot x_{m},$$

where $x_{m}$ denotes a modulation symbol drawn from *M*-ary APM constellation whose power is normalized to one and $e_{j}$ is a unit vector whose *j*th entry is non-zero for $j\in \{1,2,\cdots ,N_{t}\}$. Then, the received signal vector $y_{b}\in C^{N_{Rb}\times 1}$ at Bob and $y_{e}\in C^{N_{Re}\times 1}$ at Eve are represented by:

(2) $$y_{b}=Hx+n_{b}=h_{j}x_{m}+n_{b},$$

and:

(3) $$y_{e}=Gx+n_{e}=g_{j}x_{m}+n_{e},$$

respectively. In the above equations, $h_{j}$ and $g_{j}$ are the *j*th column vectors of **H** and **G**, respectively, and $n_{b}\in C^{N_{Rb}\times 1}$ and $n_{e}\in C^{N_{Re}\times 1}$ denote the complex additive white Gaussian noise (AWGN) vectors.

## 3. Secrecy-Enhancing Spatial Modulation Scheme

### 3.1. Antenna Selection and Insertion of AN

**Figure 2:**
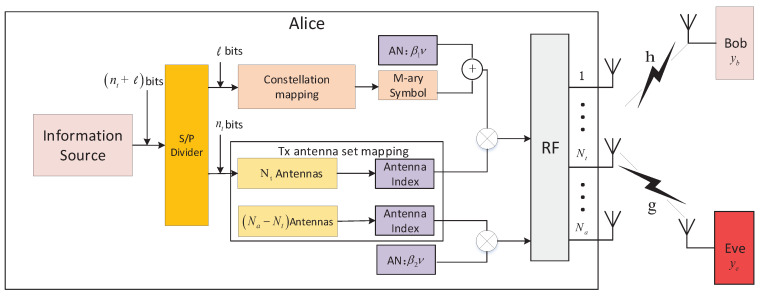
System model of the proposed secrecy enhancement scheme for the spatial modulation (SM) system.

In our proposed scheme, we consider a multiple-input single-output (MISO) system with $N_{a}$ transmit antennas as shown in Figure 2. Alice is equipped with $N_{a}$ antennas, while Bob and Eve are equipped with only one antenna. In the proposed system, we assume that $N_{a}$ is not necessarily a power of two, $N_{t}$ is a power of two, so that the antenna index can represent digital information, and $N_{a}>N_{t}$. Information symbol $x_{m}$ is transmitted along with AN of $\beta_{1}\nu$ by one of the $N_{t}$ antennas selected by matrix $T_{k}$, and another AN of $\beta_{2}\nu$ is transmitted by one of the remaining $\left(N_{a}-N_{t}\right)$ antennas selected by matrix $T_{q}$, where ν is the complex Gaussian AN with zero-mean and unit variance. In addition, $\beta_{1}$ and $\beta_{2}$ are coefficients, which are designed to cancel the AN at Bob. Accordingly, the signals received at Bob and Eve in the proposed scheme with antenna selection matrices $T_{k}$ and $T_{q}$ can be, respectively, presented by:

(4) $$\begin{array}{cc} y_{b} & =hT_{k}e_{j}\left(x_{m}+\beta_{1}\nu \right)+hT_{q}\beta_{2}\nu +n_{b}, \end{array}$$

and:

(5) $$\begin{array}{cc} y_{e} & =gT_{k}e_{j}\left(x_{m}+\beta_{1}\nu \right)+gT_{q}\beta_{2}\nu +n_{e}, \end{array}$$

where $h\in C^{1\times N_{a}}$ and $g\in C^{1\times N_{a}}$ are the row vectors representing the channel gains from Alice to Bob and Eve, respectively. $T_{k}\in C^{N_{a}\times N_{t}}$ is the effective transmit antenna selection matrix for k∈{1,2,⋯,P}, which is constructed by selecting $N_{t}$ columns from identity matrix $I_{N_{a}}$. *P* is the number of transmit antenna combinations, NaNt, and the sample space of all possible antenna combinations is represented as $\Phi =\{\Phi_{1},\cdots ,\Phi_{k},\cdots ,\Phi_{P}\}$, in which $\Phi_{k}$ denotes the *k*-th combination of the effective transmit antenna set. At each time slot, Alice selects one of the *P* combinations and shares it with Bob through a low-speed forward link, as in other conventional schemes [15]. In addition, $n_{b}$ and $n_{e}$ are complex AWGN with a mean value of zero and variance of $\sigma^{2}$. $T_{q}\in C^{N_{a}\times 1}$ is a single column matrix to select another antenna transmitting AN for $q\in \{1,2,\cdots ,N_{a}-N_{t}\}$. $T_{q}$ is selected from submatrix $I_{N_{a}}^{\prime }\in C^{N_{a}\times \left(N_{a}-N_{t}\right)}$, which is composed of the remaining column vectors in $I_{N_{a}}$ after constructing $T_{k}$.

### 3.2. Perfect Cancellation of AN

Consider the fact that perfect CSI is hard to obtain in practice due to the estimation errors. The channel gains, **h** and **g**, can be expressed by:

(6) $$\begin{array}{c} h=\sqrt{1-\rho^{2}}\hat{h}+\sqrt{\rho^{2}}\tilde{h}, \end{array}$$

and:

(7) $$\begin{array}{c} g=\sqrt{1-\rho^{2}}\hat{g}+\sqrt{\rho^{2}}\tilde{g}. \end{array}$$

Here, $\hat{h}\in C^{1\times N_{a}}$ and $\tilde{h}\in C^{1\times N_{a}}$ represent the estimation and the estimation error of the main channel, i.e., from Alice to Bob, respectively. On the other hand, $\hat{g}\in C^{1\times N_{a}}$ and $\tilde{g}\in C^{1\times N_{a}}$ represent the estimation and the estimation error of the eavesdropping channel, i.e., from Alice to Eve, respectively. $\rho^{2}$ is the variance of estimation error, and it reflects the accuracy of the CSI.

Inserting (6) into (4) leads to:

(8) $$\begin{array}{cc} y_{b}= & \sqrt{1-\rho^{2}}\hat{h}T_{k}e_{j}\left(x_{m}+\beta_{1}\nu \right)+\sqrt{\rho^{2}}\tilde{h}T_{k}e_{j}\left(x_{m}+\beta_{1}\nu \right)\\ & +\sqrt{1-\rho^{2}}\hat{h}T_{q}\beta_{2}\nu +\sqrt{\rho^{2}}\tilde{h}T_{q}\beta_{2}\nu +n_{b}. \end{array}$$

If we let ${\hat{h_{j}}}^{\prime }=\hat{h}T_{k}e_{j}$, ${\tilde{h_{j}}}^{\prime }=\tilde{h}T_{k}e_{j}$, ${\hat{h_{i}}}^{\prime }=\hat{h}T_{q}$, and ${\tilde{h_{i}}}^{\prime }=\tilde{h}T_{q}$, then:

(9) $$\begin{array}{cc} y_{b}= & \sqrt{1-\rho^{2}}{\hat{h_{j}}}^{\prime }\left(x_{m}+\beta_{1}\nu \right)+\sqrt{\rho^{2}}{\tilde{h_{j}}}^{\prime }\left(x_{m}+\beta_{1}\nu \right)\\ & +\sqrt{1-\rho^{2}}{\hat{h_{i}}}^{\prime }\beta_{2}\nu +\sqrt{\rho^{2}}{\tilde{h_{i}}}^{\prime }\beta_{2}\nu +n_{b}. \end{array}$$

Similarly, if we let ${\hat{g_{j}}}^{\prime }=\hat{g}T_{k}e_{j}$, ${\tilde{g_{j}}}^{\prime }=\tilde{g}T_{k}e_{j}$, ${\hat{g_{i}}}^{\prime }=\hat{g}T_{q}$, and ${\tilde{g_{i}}}^{\prime }=\tilde{g}T_{q}$, then (5) can be represented by:

(10) $$\begin{array}{cc} y_{e}= & \sqrt{1-\rho^{2}}{\hat{g_{j}}}^{\prime }\left(x_{m}+\beta_{1}\nu \right)+\sqrt{\rho^{2}}{\tilde{g_{j}}}^{\prime }\left(x_{m}+\beta_{1}\nu \right)\\ & +\sqrt{1-\rho^{2}}{\hat{g_{i}}}^{\prime }\beta_{2}\nu +\sqrt{\rho^{2}}{\tilde{g_{i}}}^{\prime }\beta_{2}\nu +n_{e}. \end{array}$$

Our purpose here is to make AN cancel each other at the legitimate receiver; therefore, we need to find proper values of $\beta_{1}$ and $\beta_{2}$. According to (9), we note that $\sqrt{1-\rho^{2}}{\hat{h_{j}}}^{\prime }\beta_{1}\nu +\sqrt{1-\rho^{2}}{\hat{h_{i}}}^{\prime }\beta_{2}\nu$ is the interference at Bob. Therefore, we should make it equal to zero such that there is no interference at Bob. That means:

(11) $$\sqrt{1-\rho^{2}}{\hat{h_{j}}}^{\prime }\beta_{1}\nu +\sqrt{1-\rho^{2}}{\hat{h_{i}}}^{\prime }\beta_{2}\nu =0.$$

Then, $\beta_{1}$ and $\beta_{2}$ should be calculated by:

(12) $$\left\{\begin{array}{cc} \beta_{1} & =-{\hat{h_{i}}}^{\prime }\\ \beta_{2} & ={\hat{h_{j}}}^{\prime } \end{array}\right.\;or\;\;\left\{\begin{array}{cc} \beta_{1} & ={\hat{h_{i}}}^{\prime }\\ \beta_{2} & =-{\hat{h_{j}}}^{\prime }. \end{array}\right.$$

Since the estimation channel vectors $\hat{h}$ and $\hat{g}$ are independent of each other, that is to say ${\hat{h_{j}}}^{\prime }\neq {\hat{g_{j}}}^{\prime }$ and ${\hat{h_{i}}}^{\prime }\neq {\hat{g_{i}}}^{\prime }$, $\beta_{1}$ and $\beta_{2}$ calculated in (12) will lead to:

(13) $$\sqrt{1-\rho^{2}}{\hat{g_{j}}}^{\prime }\beta_{1}\nu +\sqrt{1-\rho^{2}}{\hat{g_{i}}}^{\prime }\beta_{2}\nu \neq 0.$$

Therefore, Eve will suffer from the interference due to the AN of $\sqrt{1-\rho^{2}}{\hat{g_{j}}}^{\prime }\beta_{1}\nu +\sqrt{1-\rho^{2}}{\hat{g_{i}}}^{\prime }\beta_{2}\nu$.

### 3.3. Optimum Selection of the Transmit Antenna Combination

Assuming that the data are transmitted by the *j*-th antenna, the received signal power at Eve is $∥\hat{g}T_{k}e_{j}{∥}^{2}$, which is defined as a quantity, called leakage for Bob [16]. Generally, we can assume that the power of the received signal at Bob, i.e., $∥\hat{h}T_{k}e_{j}{∥}^{2}$, is sufficiently large compared to the power of the AWGN, (i.e., $\sigma^{2}$) and that $∥\hat{h}T_{k}e_{j}{∥}^{2}$ is sufficiently large compared to $∥\hat{g}T_{k}e_{j}{∥}^{2}$. Based on these two assumptions, we define the signal-to-leakage noise ratio (SLNR) for the *j*-th channel of the *k*-th combination as:

(14) $$\varphi_{j}\left(T_{k}\right)=\frac{∥\hat{h}T_{k}e_{j}{∥}^{2}}{∥\hat{g}T_{k}e_{j}{∥}^{2}+\sigma^{2}}.$$

Assume that the selected $N_{t}$ antennas are activated with equal probability to transmit the information symbols. The optimum matrix $T_{k}$ can be found by maximizing SLNR as follows:

(15) $$\begin{array}{c} \max\;\sum_{j=1}^{N_{t}}\varphi_{j}\left(T_{k}\right)\\ \;\mathrm{subject}\;\mathrm{to}\;T_{k}\in \left\{T_{1},T_{2},\ldots ,T_{P}\right\}. \end{array}$$

However, the above optimum solution of $T_{k}$ with exhaustive search requires a high complexity. We propose the following complexity reduced method.

Assume all of the transmit antennas are independent. Then, the SLNR at the *l*-th, $\varphi_{l}$, can be represented by:

(16) $$\varphi_{l}=\frac{∥{\hat{h}}_{l}{∥}^{2}}{∥{\hat{g}}_{l}{∥}^{2}+\sigma^{2}},$$

where ${\hat{h}}_{l}$ and ${\hat{g}}_{l}$ denote the *l*-th elements of the channel $\hat{h}$ and $\hat{g}$, respectively. In this way, we calculate the SLNR values for all transmit antennas, and the calculated SLNR values are sorted in descending order such that:

(17) $$\underset{N_{t}\;\mathrm{selected}\;\mathrm{antennas}}{\underbrace{\varphi_{\pi_{1}}\geq \varphi_{\pi_{2}}\geq \;\cdots \;\geq \varphi_{\pi_{N_{t}}}}}\geq \;\cdots \;\geq \varphi_{\pi_{N_{a}}},$$

where $\left\{\pi_{1},\pi_{2},\cdots ,\pi_{N_{a}}\right\}$ is an ordered permutation set of $\left\{1,2,\cdots ,N_{a}\right\}$. Therefore, the optimization problem in (15) is equivalent to selecting $\varphi_{\pi_{d}}$ from (17) for $1\leq d\leq N_{t}$.

## 4. Secrecy Rate Analysis

The received signal at the legitimate receiver Bob, $y_{b}$, can be rearranged as follows by inserting the result in (11) into (9).

(18) $$\begin{array}{cc} y_{b}= & \sqrt{1-\rho^{2}}{\hat{h_{j}}}^{\prime }x_{m}+\sqrt{\rho^{2}}{\tilde{h_{j}}}^{\prime }\left(x_{m}+\beta_{1}\nu \right)+\sqrt{\rho^{2}}{\tilde{h_{i}}}^{\prime }\beta_{2}\nu +n_{b}. \end{array}$$

If we let:

(19) $$\begin{array}{cc} {\tilde{n}}_{b}= & \sqrt{\rho^{2}}{\tilde{h_{j}}}^{\prime }\left(x_{m}+\beta_{1}\nu \right)+\sqrt{\rho^{2}}{\tilde{h_{i}}}^{\prime }\beta_{2}\nu +n_{b}, \end{array}$$

then it can be approximated as zero-mean complex Gaussian noise with variance of $\eta_{b}=E\left[{\tilde{n}}_{b}{\left({\tilde{n}}_{b}\right)}^{H}\right]$. In addition, letting $\theta =1-\rho^{2}$, then the normalized value of $y_{b}$ with noise variance of $\eta_{b}$ can be approximated by:

(20) $$\begin{array}{c} {\tilde{y}}_{b}=\frac{y_{b}}{\sqrt{\eta_{b}}}≃\sqrt{\frac{\theta }{\eta_{b}}}{\hat{h_{j}}}^{\prime }x_{m}+{\hat{n}}_{b}, \end{array}$$

where ${\hat{n}}_{b}={\tilde{n}}_{b}/\sqrt{\eta_{b}}$. After choosing the optimum matrices $T_{k}$ and $T_{q}$, the probability of selecting each transmit antenna and each symbol $x_{m}$ is $\frac{1}{N_{t}}$ and $\frac{1}{M}$, respectively. Thus, the received signal at Bob follows a complex Gaussian distribution, which can be represented as:

(21) $$P\left({\tilde{y}}_{b}|{\hat{h_{j}}}^{\prime },x_{m}\right)=\frac{1}{\pi }\exp\left(-|{\tilde{y}}_{b}-\sqrt{\frac{\theta }{\eta_{b}}}{\hat{h_{j}}}^{\prime }x_{m}{|}^{2}\right),$$

and:

(22) $$P\left({\tilde{y}}_{b}\right)=\frac{1}{N_{t}M}\sum_{j=1}^{N_{t}}\sum_{m=1}^{M}\frac{1}{\pi }\exp\left(-|{\tilde{y}}_{b}-\sqrt{\frac{\theta }{\eta_{b}}}{\hat{h_{j}}}^{\prime }x_{m}{|}^{2}\right).$$

Then, the ergodic rate of Bob can be written as,

(23) $$\begin{array}{c} R_{b}=E_{\hat{h}}\left[\int \sum_{j=1}^{N_{t}}\sum_{m=1}^{M}p\left({\tilde{y}}_{b},{\hat{h_{j}}}^{\prime },x_{m}\right)\log_{2}\frac{p({\tilde{y}}_{b},{\hat{h_{j}}}^{\prime },x_{m})}{p\left({\tilde{y}}_{b}\right)p\left({\hat{h_{j}}}^{\prime },x_{m}\right)}d{\tilde{y}}_{b}\right]\\ =\log_{2}N_{t}M-\frac{1}{N_{t}M}\sum_{j=1}^{N_{t}}\sum_{m=1}^{M}E_{\hat{h}}\left[E_{{\hat{n}}_{b}}\left[\log_{2}\sum_{j^{\prime }=1}^{N_{t}}\sum_{m^{\prime }=1}^{M}\right.\right.\\ \;\;\;\times \left.\left.\exp\left(-\left(|\sqrt{\frac{\theta }{\eta_{b}}}\delta_{j,m}^{j^{\prime },m^{\prime }}+{\hat{n}}_{b}{|}^{2}-|{\hat{n}}_{b}{|}^{2}\right)\right)\right]\right], \end{array}$$

in which $\delta_{j,m}^{j^{\prime },m^{\prime }}$ is given by:

(24) $$\delta_{j,m}^{j^{\prime },m^{\prime }}={\hat{h_{j}}}^{\prime }x_{m}-{\hat{h_{j^{\prime }}}}^{\prime }x_{m^{\prime }}.$$

Using Jensen’s inequality, the lower bound of $R_{b}$ is obtained as:

(25) $$\begin{array}{cc} R_{b}^{low} & =\log_{2}N_{t}M-\frac{1}{N_{t}M}\sum_{j=1}^{N_{t}}\sum_{m=1}^{M}\log_{2}\sum_{j^{\prime }=1}^{N_{t}}\sum_{m^{\prime }=1}^{M}\\ & \;\;\;\times E_{\hat{h}}\left[E_{{\hat{n}}_{b}}\left[\exp\left(-\left(|\sqrt{\frac{\theta }{\eta_{b}}}\delta_{j,m}^{j^{\prime },m^{\prime }}+{\hat{n}}_{b}{|}^{2}-|{\hat{n}}_{b}{|}^{2}\right)\right)\right]\right]\\ & =\log_{2}N_{t}M+1-\frac{1}{\ln2}\\ & \;\;\;-\frac{1}{N_{t}M}\sum_{j=1}^{N_{t}}\sum_{m=1}^{M}\log_{2}\sum_{j^{\prime }=1}^{N_{t}}\sum_{m^{\prime }=1}^{M}E_{\hat{h}}\left[\exp\left(-\frac{\theta |\delta_{j,m}^{j^{\prime },m^{\prime }}{|}^{2}}{2\eta_{b}}\right)\right]. \end{array}$$

In order to derive the ergodic rate at the passive eavesdropper, we first represent (10) using θ as follows:

(26) $$\begin{array}{cc} y_{e} & =\sqrt{\theta }{\hat{g_{j}}}^{\prime }x_{m}+\\ & \sqrt{\theta }\left({\hat{g_{j}}}^{\prime }\beta_{1}+{\hat{g_{i}}}^{\prime }\beta_{2}\right)\nu +\sqrt{\rho^{2}}\left({\tilde{g_{j}}}^{\prime }x_{m}+{\tilde{g_{j}}}^{\prime }\beta_{1}\nu +{\tilde{g_{i}}}^{\prime }\beta_{2}\nu \right)+n_{e}. \end{array}$$

If we let:

(27) $$\begin{array}{cc} {\tilde{n}}_{e}= & \sqrt{\theta }\left({\hat{g_{j}}}^{\prime }\beta_{1}+{\hat{g_{i}}}^{\prime }\beta_{2}\right)\nu +\sqrt{\rho^{2}}\left({\tilde{g_{j}}}^{\prime }x_{m}+{\tilde{g_{j}}}^{\prime }\beta_{1}\nu +{\tilde{g_{i}}}^{\prime }\beta_{2}\nu \right)+n_{e}, \end{array}$$

then the normalized value of $y_{e}$ with $\eta_{e}=E\left[{\tilde{n}}_{e}{\left({\tilde{n}}_{e}\right)}^{H}\right]$ can be approximated by:

(28) $$\begin{array}{c} {\tilde{y}}_{e}≃\sqrt{\frac{\theta }{\eta_{e}}}{\hat{g_{j}}}^{\prime }x_{m}+{\hat{n}}_{e}, \end{array}$$

where ${\hat{n}}_{e}={\tilde{n}}_{e}/\sqrt{\eta_{e}}$.

Then, the ergodic rate of Eve is expressed as:

(29) $$\begin{array}{c} R_{e}=E_{\hat{g}}\left[\int \sum_{j=1}^{N_{t}}\sum_{m=1}^{M}p\left({\tilde{y}}_{e},{\hat{g_{j}}}^{\prime },x_{m}\right)\log_{2}\frac{p({\tilde{y}}_{e},{\hat{g_{j}}}^{\prime },x_{m})}{p\left({\tilde{y}}_{e}\right)p\left({\hat{g_{j}}}^{\prime },x_{m}\right)}d{\tilde{y}}_{e}\right]\\ =\log_{2}N_{t}M-\frac{1}{N_{t}M}\sum_{j=1}^{N_{t}}\sum_{m=1}^{M}E_{\hat{g}}\left[E_{{\hat{n}}_{e}}\left[\log_{2}\sum_{j^{\prime }=1}^{N_{t}}\sum_{m^{\prime }=1}^{M}\right.\right.\\ \;\;\;\times \left.\left.\exp\left(-\left(|\sqrt{\frac{\theta }{\eta_{e}}}\alpha_{j,m}^{j^{\prime },m^{\prime }}+{\hat{n}}_{e}{|}^{2}-|{\hat{n}}_{e}{|}^{2}\right)\right)\right]\right], \end{array}$$

in which $\alpha_{j,m}^{j^{\prime },m^{\prime }}$ is given by:

(30) $$\alpha_{j,m}^{j^{\prime },m^{\prime }}={\hat{g_{j}}}^{\prime }x_{m}-{\hat{g_{j^{\prime }}}}^{\prime }x_{m^{\prime }}.$$

Using Jensen’s inequality, the lower bound of $R_{e}$ is obtained as:

(31) $$\begin{array}{c} R_{e}^{low}=\log_{2}N_{t}M+1-\frac{1}{\ln2}\\ \;\;\;-\frac{1}{N_{t}M}\sum_{j=1}^{N_{t}}\sum_{m=1}^{M}\log_{2}\sum_{j^{\prime }=1}^{N_{t}}\sum_{m^{\prime }=1}^{M}E_{\hat{g}}\left[\exp\left(-\frac{\theta |\alpha_{j,m}^{j^{\prime },m^{\prime }}{|}^{2}}{2\eta_{e}}\right)\right]. \end{array}$$

Based on (23) and (29), the ergodic secrecy rate can be written as:

(32) $${\bar{R}}_{s}=\max\left\{0,R_{b}-R_{e}\right\}.$$

## 5. Simulation and Numerical Results

This section presents the secrecy performance comparisons in terms of the secrecy rate. The ergodic secrecy rate, derived in (32), was numerically simulated by using several parameters including $N_{a}$, $N_{t}$, modulation order *M*, and SNR values. In the simulations, the power of the modulation symbol was normalized to one, and the power of the complex AWGN at both Bob and Eve was assumed to be $\sigma^{2}$, while the SNR was $1/\sigma^{2}$.

**Figure 3:**
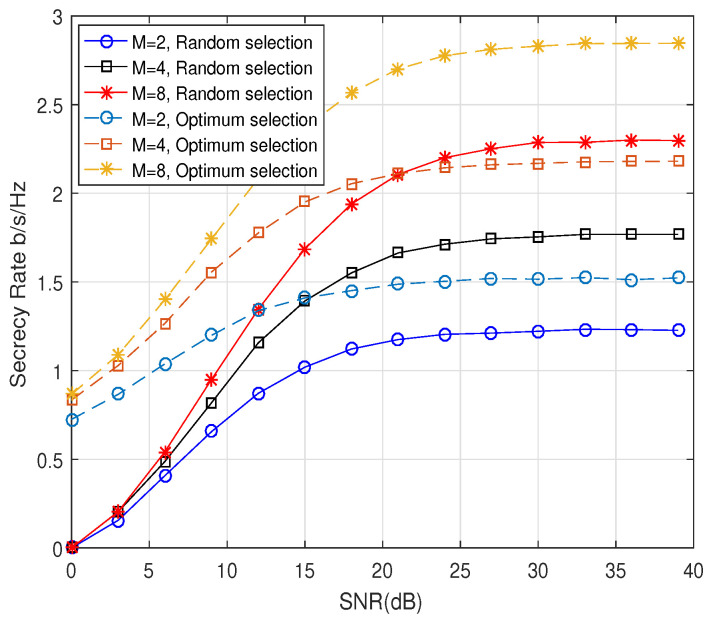
The secrecy rate, ${\bar{R}}_{s}$, of the proposed scheme according to the selection methods of $T_{k}$ when $N_{a}=6$ and $N_{t}=4$.

We first compared the secrecy performance of the proposed scheme according to the antenna selection method. Figure 3 shows the comparison of the secrecy performance when Alice chooses $T_{k}$ by using the optimum selection method described in Section 3.3 and the random selection method, when $\rho^{2}$ = 0.01. It was assumed that $N_{a}$ = 6 and $N_{t}$ = 4, and different APM schemes with *M* = 2, *M* = 4, and *M* = 8 were employed. As shown in Figure 3, compared to the random selection scheme, the proposed optimum antenna selection scheme with $T_{k}$ was able to achieve a better secrecy rate. Because the secrecy capacity of fading channels had a ceiling as the transmit SNR increased [17], ${\bar{R}}_{s}$ of the proposed scheme increased with the increasing SNR until the SNR approached about 30 dB. Furthermore, it is easy to see that a higher modulation order *M* yielded a higher secrecy rate.

**Figure 4:**
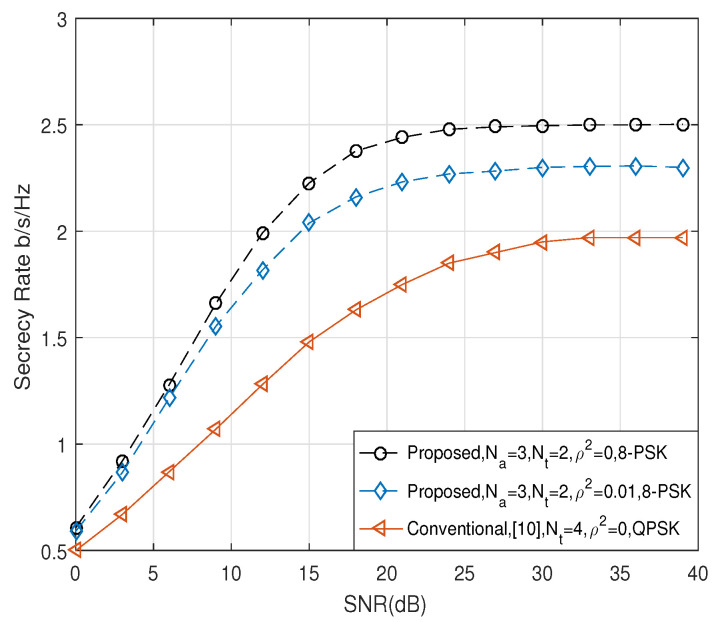
Comparison of ${\bar{R}}_{s}$ when $r_{e}$ = 4 bits.

**Figure 5:**
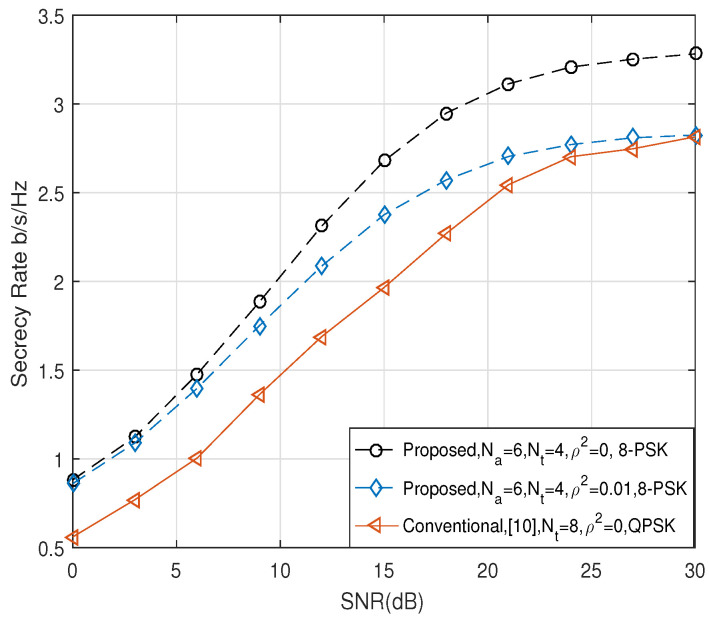
Comparison of ${\bar{R}}_{s}$ when $r_{e}$ = 5 bits.

Next, we compared the secrecy rate of the proposed scheme with the conventional scheme in [9], by applying the same effective data rate, $r_{e}=n_{t}+\ell$. Figure 4 and Figure 5 show the comparison of ${\bar{R}}_{s}$ when $r_{e}$ were four and five, respectively. When $r_{e}=4$ in Figure 4, we considered the scenario that Alice employed the 8-PSK modulation, and $N_{a}$ and $N_{t}$ were set to three and two, respectively, in the proposed scheme. On the other hand, in the conventional scheme, Alice employed QPSK modulation, and the symbol was transmitted by one of four transmit antennas, i.e., $N_{t}=4$. When $r_{e}=5$ in Figure 5, $N_{a}$ and $N_{t}$ of the proposed scheme were set to be six and 4, respectively, and the 8-PSK modulation symbol was transmitted. On the other hand, $N_{t}$ of the conventional scheme was set to be eight, and QPSK symbol was transmitted. As shown in Figure 4 and Figure 5, we observe that the proposed scheme can achieve better secrecy performance than the conventional scheme in [9] when the perfect CSI was given, i.e., $\rho^{2}=0$. Furthermore, the proposed scheme with channel estimation error of $\rho^{2}=0.01$ produced a better performance compared to the conventional scheme with perfect CSI.

**Figure 6:**
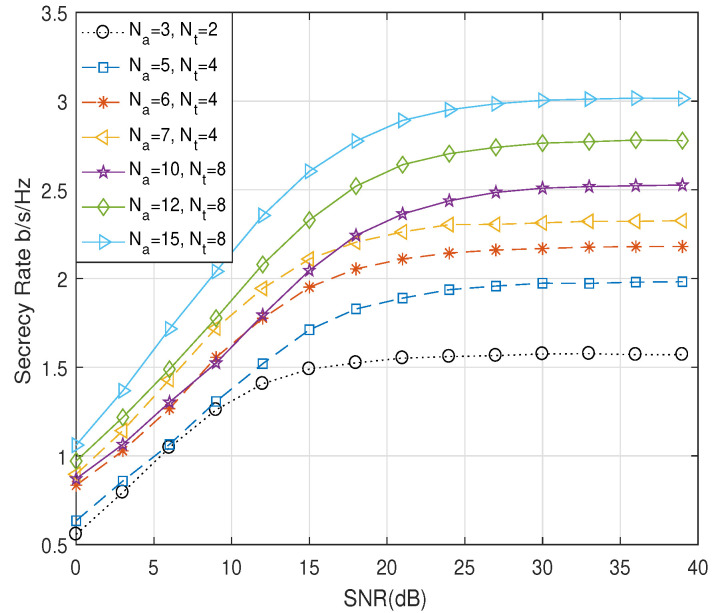
Comparison of ${\bar{R}}_{s}$ for the proposed scheme according to $N_{a}$ and $N_{t}$.

Figure 6 demonstrates the performance of the secrecy rate of the proposed scheme versus SNR with different numbers of transmit antennas $N_{a}$ and $N_{t}$, when $\rho^{2}$ was equal to 0.01. We applied the QPSK modulation. We assumed that Alice was equipped with $N_{a}$ = 3, 5, 6, 7, 10, 12, and 15 transmit antennas and utilized $N_{t}$ = 2, 4, and 8 effective transmit antennas to send information. As shown in Figure 6, with the increase in the number of transmit antennas $N_{t}$, the secrecy rate was remarkably improved. When we set $N_{t}$ to be a fixed value, the more antennas $N_{a}$ Alice had, the higher the secrecy rate was. Simulation results also showed that the secrecy rate was growing rapidly under the low SNR. As the SNR increased, the secrecy rate tended to approach a saturation point. Because in the case of the low SNR, most of the transmit power was used to transmit the information symbols, more transmit power was used to transmit the AN in order to improve the secrecy rate as the SNR increased.

**Figure 7:**
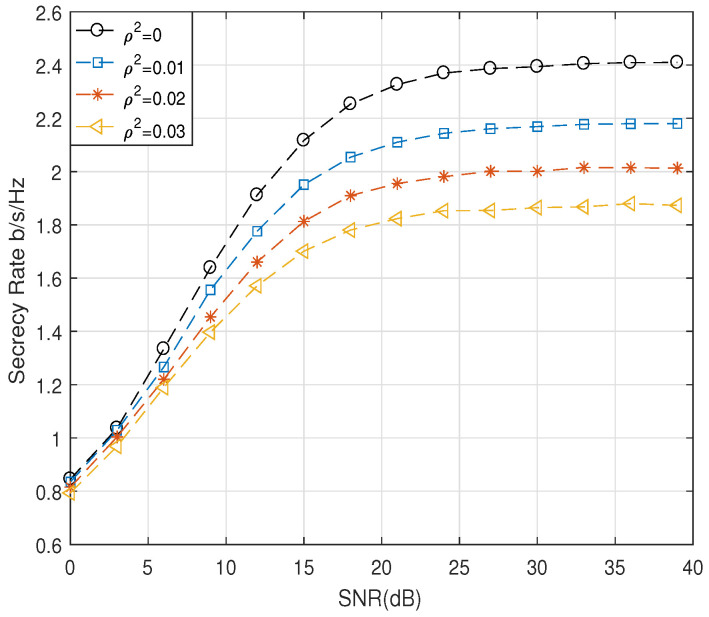
Comparison of ${\bar{R}}_{s}$ for the proposed scheme with varying $\rho^{2}$.

**Figure 8:**
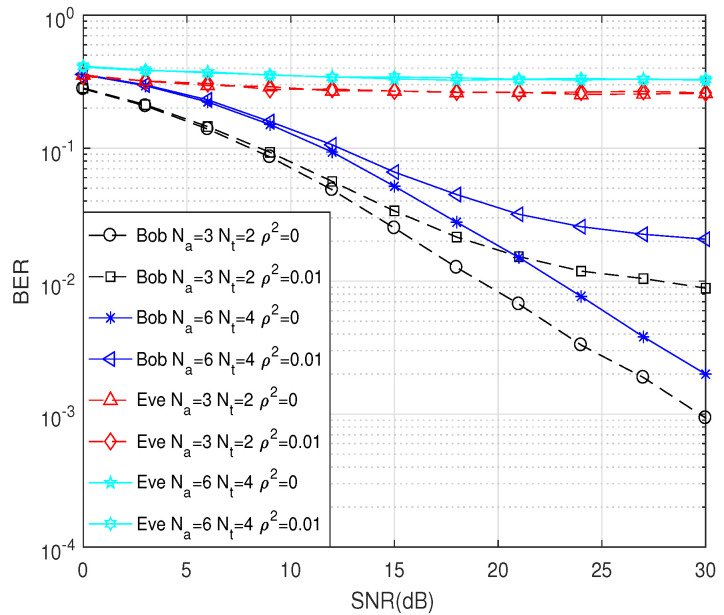
The BER performance under BPSK modulation and various numbers of $N_{a}$ and $N_{t}$.

Figure 7 shows the secrecy rate against the SNR with varying channel estimation error, $\rho^{2}$, where $N_{a}$ and $N_{t}$ were set to six and four, respectively. As we can easily deduce, the secrecy rate decreased as $\rho^{2}$ increased. Finally, Figure 8 compares the bit error rate (BER) performances of the proposed scheme. We employed the BPSK modulation scheme and various numbers of $N_{a}$ and $N_{t}$ at Alice. It was shown that the BER performance at Bob decreased rapidly with SNR for varying estimation error. However, BER at Eve was nearly 0.5 for all SNRs, which indicated that there was no information leaked to Eve.

## 6. Conclusions

In this paper, we presented a new secrecy-enhancing scheme for the SM system. The proposed scheme employed two AN sequences, which were designed to be canceled each other perfectly only at the legitimate receiver. The AN sequences were designed by exploiting imperfect CSI of the legitimate channel by considering a practical system environment. In addition, an optimum antenna selection scheme was proposed by reducing the complexities of finding the solution from the exhaustive search. With the proposed scheme, the passive eavesdropper would suffer from the interference induced by the AN, resulting in secrecy rate enhancement of the legitimate receiver. We analyzed the secrecy performance over a Rayleigh fading channel by considering imperfect CSI. The simulation results demonstrated that the secrecy performance of the proposed scheme was enhanced compared to the conventional scheme.
